# Single-Input Regulatory Cascade for *in vivo* Removal of the Solubility Tag in Fusion Recombinant Proteins Produced by *Escherichia coli*

**DOI:** 10.3389/fbioe.2019.00200

**Published:** 2019-08-20

**Authors:** Filipe S. R. Silva, Sara P. O. Santos, Roberto Meyer, Neuza M. Alcantara-Neves, Carina S. Pinheiro, Luis G. C. Pacheco

**Affiliations:** Post-graduate Program in Biotechnology, Institute of Health Sciences, Federal University of Bahia, Salvador, Brazil

**Keywords:** recombinant proteins, protein solubility, synthetic biology, *Escherichia coli*, green fluorescent protein

## Abstract

Solubility tags are commonly fused to target recombinant proteins to enhance their solubility and stability. In general, these protein tags must be removed to avoid misfolding of the partner protein and to allow for downstream applications. Nevertheless, *in vitro* tag removal increases process complexity and costs. Herein, we describe a synthetic biology-based strategy to permit *in vivo* removal of a solubility tag (EDA, KDPG aldolase), through co-expression of the fusion recombinant protein (EDA-EGFP) and the tag-cleaving protease (TEVp), in a controlled manner. Basically, the system uses three repressor proteins (LacI, cI434, and TetR) to regulate the expressions of EDA-EGFP and TEVp, in a regulatory cascade that culminates with the release of free soluble target protein (EGFP), following a single chemical induction by IPTG. The system worked consistently when all biological parts were cloned in a single plasmid, pSolubility(SOL)A (7.08 Kb, Amp^R^), and transformed in *Escherichia coli* Rosetta (DE3) or BL21(DE3) strains. Total soluble recombinant protein yield (EDA-EGFP + free EGFP) was *ca*. 272.0 ± 60.1 μg/mL of culture, following IMAC purification; free EGFP composed great part (average = 46.5%; maximum = 67.3%) of the total purified protein fraction and was easily separated from remaining fusion EDA-EGFP (53 KDa) through filtration using a 50 KDa cut-off centrifugal filter.

## Introduction

Fusion protein tags are normally used for successfully obtaining hard-to-express recombinant proteins in their soluble form in bacteria. A fusion tag can enhance a given recombinant protein quality by improving its translation, avoiding protein aggregation and even shielding it from degradation (Waugh, 2005; Kang et al., 2015; Bernier et al., 2018). Commonly used solubility enhancers include Maltose-binding protein (MBP, 42.5 KDa), Glutathione-S-transferase (GST, 26 KDa), Thioredoxin A (TrxA, 12 KDa), and N-utilization substance protein A (NusA, 55 KDa). Following expression of the fused recombinant protein, these protein tags need to be detached as they can significantly affect a given passenger protein's biological function. For this, specific protease cleavage sites are placed in between the fusion tag and the target protein, which can then be recovered in its natural form after *in vitro* incubation with the respective proteases, such as the Tobacco Etch Virus protease (TEVp), followed by chromatographic steps. However, these post-processing steps increase production costs and process intricacy (Li, 2011). To circumvent these technical difficulties, some studies have tried to co-express the specific protease with the fusion protein to get the unfused target protein *in vivo* in a simpler manner (Kapust and Waugh, 2000; Shih et al., 2005; Wei et al., 2012; Feng et al., 2014; Luo et al., 2015). Generally, co-expression of TEVp with the fusion target protein is done by using different inducing agents (e.g., IPTG and aTc) (Kapust and Waugh, 2000), or by using the same operator site to control transcription of both genes (Wei et al., 2012). The protease can also be constitutively expressed through chromosomal integration, or transcriptionally fused to the cassette that codes for the fusion protein (Shih et al., 2005).

**Figure 1:**
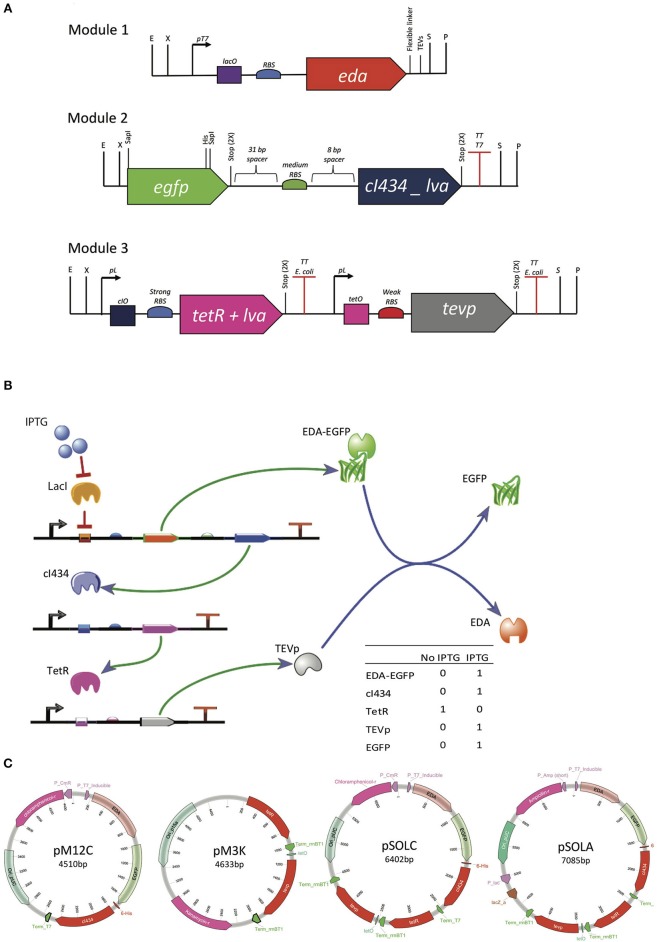
Genetic organization of the system for controlled intracellular processing of recombinant proteins. **(A)** Genetic modules built with biological parts described in Supplementary Table S1, synthetized with RFC23 Biobrick standard, to allow for easy assembly. **(B)** Genetic circuit graphic simulation, built with TinkerCell (Chandran et al., 2009). **(C)** Plasmids assembled from the tree different modules. Modules were distributed in two different plasmids (pM12C + pM3K) or joined in one plasmid (pSOLA or pSOLC). pM12C contains both modules 1 and 2 joined together and has pSB1C3 (high copy, Cm^R^) backbone. pM3K has the module 3 in a pSB1K3 (low copy, Km^R^) backbone. pSOLC includes all three modules inserted in pSB1C3 (high copy, Cm^R^) and pSOLA holds all three modules introduced in pUC57 backbone (high copy, Amp^R^).

In this brief report, we propose a strategy based on a regulatory cascade to produce both the target fusion protein and the tag-cleaving protease TEVp through a single chemical induction, using different operator sites. Similarly, to the repressilator genetic circuit (Elowitz and Leibler, 2000), our system uses three repressor proteins (LacI, cI434, and TetR) to regulate the expression of the target fusion protein and the TEVp, in a regulatory cascade that culminates with *in vivo* release of EGFP from its solubility tag (Figures 1A,B).

## Materials and Methods

### Genetic Circuit Design and Biological Parts Selection

The genetic elements used to compose the three genetic modules shown in Figure 1A were retrieved from the iGEM Registry of Standard Biological Parts (http://parts.igem.org/Main_Page) and from selected previous studies (Supplementary Table S1). The first module contains the T7 promoter, the *lacO* operator site and an RBS derived from the registry part # BBa_K567018. The sequence coding for a fusion target protein consisting of the solubility tag KDPG aldolase (EDA), a Gly-Ser-Gly-Ser flexible linker, a canonical TEVp cleavage recognition site (Glu-Asn-Leu-Tyr-Phe-Gln↓Gly) and EGFP, was then put under control of these genetic elements (Figure 1A). A 31 bp spacer sequence was placed upstream and an 8 bp spacer was situated downstream a medium strength RBS, which controls the translation of the cI434 repressor, that is transcriptionally coupled to the sequence encoding the fusion protein. The third module was designed to express the TetR repressor under control of the lambda promoter sequence, which is regulated by the cI434 repressor (Figure 1A). This way, TetR is expected to be produced when IPTG is absent in the growth medium (Figure 1B). Lastly, the TEVp is produced under the control of a TetR regulated promoter and translated using a weak RBS (Figure 1A). The repressor proteins have a C-terminal LVA degradation tail, which is expected to expedite degradation of these regulators in *Escherichia coli*, in order to prevent the circuit from collapsing due to the accumulation of regulators (Brophy and Voigt, 2014) (Supplementary Figure S1A).

### Plasmids Design and Construction

The three modules were designed containing RFC23 BioBricks™ standard sites at extremities in order to facilitate assembling (Figure 1A) (Røkke et al., 2014). Synthetic constructions were purchased from GenScript (Scotch Plains, NJ, USA), initially cloned in pUC57 and then sub-cloned in BioBricks compatible plasmid backbones (Supplementary Table S2). Module 2 was isolated from pM2A vector by digesting it with *EcoRI* and *PstI* enzymes. Then, it was inserted into the predigested BioBricks compatible plasmid pSB1C3 to generate pM2C. To connect modules 1 and 2, Silver assembly (Phillips and Silver, 2006) was performed to join together EDA and EGFP coding sequences. For this, pM1A containing the EDA coding sequence was digested with *EcoRI* and *SpeI*, releasing the module 1 fragment (Figure 1A). On the other hand, pM2C was linearized with *EcoRI* and *XbaI*. The isolated module 1 and pM2C fragments were joined together using T4 DNA ligase (Promega), and the resulting plasmid was named pM12C. pM12C was then linearized with *SpeI* and *PstI* and pM3A was cut with *XbaI* and *PstI*. Following purification, these two fragments were ligated to the form pSOLC, which contains the three modules. pSOLC was digested with *EcoRI* and *PstI* and then inserted back in pUC57, resulting in the plasmid pSOLA. Finally, pM3A was also digested with *EcoRI* and *PstI* and cloned into pSB1K3 to give pM3K (http://partsregistry.org/Part:pSB1C3). Plasmids constructions are summarized in Figure 1C. Details are given on Supplementary Methods.

### Recombinant Protein Expression, Purification, and Analysis

Chemically transformed *E. coli s*trains (BL21, Rosetta™, and CodonPlus-RIL), were routinely maintained at 37°C, with aeration, in Luria-Bertani (LB) broth or LB-agar plates, containing the appropriate antibiotics according to the plasmid-conferred resistances (pSOLA/Amp^R^; pSOLC/Cm^R^; pM12C + pM3K/Cm^R^ + Kan^R^). Recombinant protein production was induced by the addition of 0.5 mM IPTG to growth media, when cells reached optical densities (at 600 nm) of 0.6, 1.5, or 3.0; bacterial cultures were further incubated at 25°C for up to 24 h. Fluorescence emission by recombinant expression of EGFP in cultures was monitored by Fluoroskan Ascent™ Microplate Fluorimeter (Ex. = 485 nm; Em. = 535 nm). Aliquots were collected at different time points, bacterial pellets were lysed by sonication in FastBreak™ Cell Lysis Reagent (Promega, Madison, WI, USA), and total protein extracts were analyzed by 12% SDS-PAGE (250 mM of DTT or BME) and Western blotting using eGFP Tag Monoclonal Antibody (Invitrogen, F56-6A1.2.3, 1:4000). IMAC protein purification of 6xHis-tagged recombinant proteins was performed using MagneHis™ (Promega, Madison, WI, USA). Additionally, the recovered purified protein fraction was filtered through Amicon™ (Lexington, MA, USA) Ultra 2 mL Centrifugal Filter (50 kDA cut-off) (see Supplementary Methods for details).

## Results and Discussion

Figure 1 shows the genetic organization of the system for controlled intracellular processing of a recombinant fusion protein, in order to release the solubility tag *in vivo* with a single chemical induction. The expected functioning of the system is the following: upon IPTG induction, the target fusion protein (EDA-EGFP) is produced along with the cI434 repressor; cI434 in turn binds to its cognate operator site and stops TetR production; TEV protease, which is repressed by binding of TetR to *tetO* operator site, then starts to be produced (Figure 1B; Supplementary Figure S1A). The genetic modules were all cloned in a single plasmid (pSOLA or pSOLC, for Amp^R^ and Cm^R^, respectively) or in two different plasmids (pM12C + pM3K, Cm^R^ and Kan^R^), with differing copy numbers, in order to tune the production of the various components at their required levels (Figures 1A,C; Supplementary Table S2).

**Figure 2:**
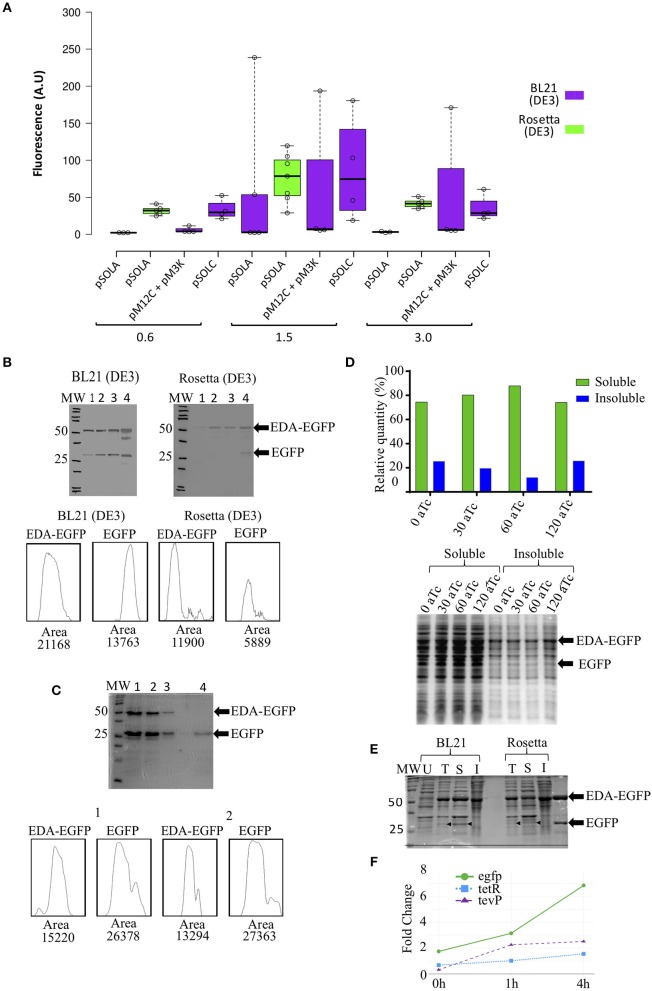
EDA-EGFP fusion protein production and levels of released EGFP, using different genetic organizations and *E. coli* strains. **(A)** Fluorescence measurements after 24 h of IPTG induction at different optical densities (OD_600nm_); **(B)** Western blot detection of EDA-EGFP and EGFP proteins by anti-eGFP Tag Monoclonal Antibody at: (1) 0 h, (2) 4 h, (3) 6 h, and (4) 24 h post induction. Densitometric analyses of Western blot detections (lane 4) is presented; 30 μg of each sample was loaded per each well; **(C)** 15% SDS-PAGE from protein purification of untagged EGFP. Below are densitometric analysis of 24 h protein profile for both BL21 and Rosetta by ImageJ software. (1 and 2) 6xHis-tagged proteins obtained following IMAC purification (MagneHis™ Protein Purification System, soluble protein protocol); (3) Retained protein concentrate in the filter Amicon™ Ultra (>50 KDa); (4) collected EGFP fraction (flow-through) (<50 KDa). Densitometric analysis by ImageJ of lanes 1 and 2 are also shown. **(D)** Relative quantification of EGFP found in the soluble and insoluble fractions (from cultures containing 0, 30, 60, and 120 ng/mL of anhydrotetracycline) in SDS-PAGE after solubility test. **(E)** 12% SDS-PAGE of (U) uniduced cells; (T) total lysated cells and (S) soluble and (I) insoluble fractions from solubility test. Thirty microgram of total proteins were loaded in each lane. **(F)** Relative gene expression analysis of the three transcriptional units in Rosetta(DE3) at 0, 1, and 4 h after induction with IPTG. Primers used are listed in Supplementary Table S4.

BL21(DE3) *E. coli* cells carrying pSOLA (which has the three genetic modules in a single plasmid) rendered the highest EGFP fluorescence signal among all tested conditions, when IPTG induction was added at an OD_600nm_ = 1.5 (mean fluorescence units FU = 60.01 ± 102.30 A.U.; maximum FU = 238.70 A.U), though fluorescence levels were highly variable in this strain (Figure 2A); significant increase in recombinant protein expression was reached at 24 h post-induction in this strain (Supplementary Figure S2). *E. coli* Rosetta (DE3) in turn showed a more reproducible EGFP fluorescence signal generation throughout all replicates, despite reaching apparently lower induction levels (mean FU = 76.09 ± 32.89 A.U.; maximum FU = 119.50 A.U.) (Figure 2A); noteworthy, these fluorescence values were not significantly different from other induction conditions at OD_600nm_ = 1.5, indicating that the system works similarly in both strains (Figure 2A; Supplementary Table S3 and Figure S2). Rosetta (DE3) can be used to overcome low yield and poor solubility of recombinant TEVp produced in *E. coli* (Wei et al., 2012; Cesaratto et al., 2016). We hypothesize that this might be a contributing factor for obtaining more predictable results using this strain in this study.

While EGFP accumulation was also observed in the two-plasmid based system (pM12C + pM3K) using *E. coli* BL21 (DE3), no TEVp activity was detected *in vivo* (not shown); conversely, the fused EDA-EGFP (53 KDa) protein was completely cleaved *in vitro* with purified recombinant TEVp, releasing the EDA tag (23 KDa) and the his-tagged recombinant EGFP (30 KDa) (Supplementary Figure S2D). The single-plasmid based system (pSOLA) in turn, showed significant *in vivo* cleavage of the EDA-EGFP fusion protein for both BL21 (DE3) and Rosetta (DE3) (Figure 2B). Significant leaking is observed when EDA-EGFP is expressed in BL21(DE3); besides, released EGFP can be found at early induction times, but accumulates at higher concentrations at 24 h post-induction. Conversely, expression in Rosetta (DE3) was closer to what would be expected from the genetic system functioning (Figure 2B); this is also confirmed by gene expression analysis of the three transcriptional units that compose the system, which shows higher expression of *egfp* when compared to *tevP*, in all time points (Figure 2F; Supplementary Figure S1B). Novel combinations of biological parts can be tested in future constructions to evaluate their effects on fine-tuning of the genetic system. This will be important to address a limitation of our approach, that was the persistence of significant part of the recombinant protein still in its fusion form *in vivo* (Figures 2B,E), whereas previous studies of controlled intracellular processing in *E. coli* have achieved almost complete processing of solubility tags (Kapust and Waugh, 2000; Nallamsetty et al., 2004; Raran-kurussi and Waugh, 2016).

Soluble 6xHis-tagged proteins were purified using a bead-based protocol, yielding *ca*. 272.0 ± 60.1 μg/mL of purified recombinant EDA-EGFP and free EGFP per mL of culture (Figure 2C; Supplementary Figure S2). Densitometric analysis showed variable proportions of EDA-EGFP/EGFP, ranging from 0.3- to 2-fold concentration of untagged protein compared to EDA-tagged protein (Figure 2C; Supplementary Figure S2). Purified protein was then submitted to diafiltration using a 50 KDa cut-off centrifugal filter, in order to separate fusion EDA-EGFP from detached EGFP (Figure 2C; Supplementary Figure S2). In future configurations, EDA can be substituted by another solubility partner such as MBP, then permitting removal by affinity chromatography (Kosobokova et al., 2016).

Increasing concentrations of anhydrotetracycline (aTc) were added to the culture media after 4 h of IPTG induction to check whether it would enhance *in vivo* protein cleavage, as described by Kapust and Waugh (2000). The proportion of soluble EGFP recovered was around 80.0% of total recombinant EGFP protein produced, either with no addition of aTc or with aTc concentrations ranging from 30 to 120 ng/mL (Figure 2D). These results indicate that only IPTG induction is sufficient to simultaneously express the fusion protein and TEVp, resulting in untagged EGFP in absence of anhydrotetracycline. Figure 2E shows that released EGFP is found mostly in the soluble fraction.

The genetic regulatory cascade described here is composed by genetic elements that interact among themselves resulting in the simultaneous production of a fusion recombinant protein and of the site-specific protease that separates the solubility tag from the target protein, all with a single induction. The main characteristics of this genetic system are: (i) it requires only a single inducing agent (IPTG); (ii) it is tuned to produce a higher amount of the fusion recombinant protein than the tag-cleaving protease; (iii) it can potentially be adapted to any cell lineage that produces T7 RNA polymerase. This genetic circuit is able to perform the task of co-producing both EDA-EGFP fusion protein with tag-cleaving TEVp, then resulting in an average of 46.5% (maximum 67.3%) of soluble EGFP release *in vivo* (Figure 2C).

## Author Contributions

FS and SS conducted all the experiments. RM, NA-N, CP, and LP conceived experiments, discussed results, and contributed to manuscript writing. All authors read and corrected the final manuscript.

### Conflict of Interest Statement

The authors declare that the research was conducted in the absence of any commercial or financial relationships that could be construed as a potential conflict of interest.

## Abbreviations

aTc: anhydrotetracycline
BCIP: 5-Bromo-4-chloro-3-indolyl phosphate
BME: 2-mercaptoethanol
cI434: phage 434 repressor protein
*cI434O*: operator site repressible by cI434
DTT: dithiothreitol
EDA: KHG/KDPG aldolase
EGFP: enhanced green fluorescent protein
iGEM: International Genetically Engineered Machine
IMAC: Immobilized metal affinity chromatography
IPTG: Isopropyl β-D-1-thiogalactopyranoside
KDPG: 2-Keto-3-deoxy-6-phosphogluconate
LVA: leucine - valin - alanine
NBT: nitroblue tetrazolium
OD: optical density
RBS: ribosome biding site
RFC23: BioBrick™ request for comments 23/Silver assembly
TEV: Tobacco Etch Virus.
