# Supplementary material for: Single-Input Regulatory Cascade for in vivo Removal of the Solubility Tag in Fusion Recombinant Proteins Produced by Escherichia coli
Source: Front Bioeng Biotechnol. 2019 Aug 20;7:200. doi: 10.3389/fbioe.2019.00200 (PMC6710347; doi:10.3389/fbioe.2019.00200)
Supplement: Supplementary file 1 [file Data_Sheet_1.docx]

**Supplementary Material**

**Single-Input Regulatory Cascade for *In Vivo* Removal of the Solubility Tag in Fusion Recombinant Proteins Produced by *Escherichia coli***

Filipe S. R. Silva, Sara P. O. Santos, Roberto Meyer, Neuza M. Alcantara-Neves, Carina S. Pinheiro, Luis G. C. Pacheco *

*Post-Graduate Program in Biotechnology, Institute of Health Sciences, Federal University of Bahia, Salvador-BA, Brazil.*

*** Correspondence:** luis.pacheco@ufba.br

**Supplementary Table S1** – Full description of biological parts used to compose the genetic circuit.

| **Part** | **Description** | **Source** |
| --- | --- | --- |
| *eda* | Coding sequence for *E. coli* 2-Keto-3-deoxy-6-phosphogluconate (KDPG) Aldolase. | (Kang et al., 2015) |
| *pT7* | T7 bacteriophage strong promoter derivative. | Registry of Standard Biological Parts (iGEM Foundation): Part BBa_K567018; designed by Xiaopan Ma e Xiwen Zhao. Available in: http://parts.igem.org/Part:BBa_K567018 |
| *lacO* | Lac operon operator site. |  |
| RBS | Ribosome Biding Site |  |
| *egfp* | EGFP encoding gene. |  |
| *Flexible linker* | Flexible protein domains linker GSGS. | (Correa et al., 2014)  (Correa et al., 2014) |
| TEV site (*TEVs*) | Canonic TEV protease cleaving site. |  |
| *pL-cIO* | Modified lambda promoter regulated by repressor protein cI 434. | Registry of Standard Biological Parts (iGEM Foundation): Part BBa_I12006, designed by Mcnamara. Available in: http://parts.igem.org/Part:BBa_I12006. |
| *pL-tetO* | Hybrid promoter composed by lambda phage promoter (pL) and *tetO* operator site. | Registry of Standard Biological Parts (iGEM Foundation): Part BBa_R0040, designed by June Rhee, Connie Tao, Ty Thomson e Louis Waldman. Available in: http://parts.igem.org/Part:BBa_R0040. |
| *31 bp spacer* | Sequences Upstream of the Ribosome that allows suitable transcription of two genes by single promoter. | (Lentini et al., 2013) |
| *8 bp spacer* | Sequences Upstream of the Ribosome that allows suitable transcription of two genes by single promoter. | (Lentini et al., 2013) |
| *weak RBS* | Ribosome biding site with low affinity for *E. coli* ribosomal 16S RNA. | Registry of Standard Biological Parts (iGEM Foundation): Part BBa_B0033, designed by Vinay S Mahajan, Voichita D. Marinescu, Brian Chow, Alexander D Wissner-Gross e Peter Carr. Available in: http://parts.igem.org/Part:BBa_B0033. |
| *medium RBS* | Ribosome biding site with medium affinity for *E. coli* ribosomal 16s RNA. | Registry of Standard Biological Parts (iGEM Foundation): Part BBa_B0032, designed by Vinay S Mahajan, Voichita D. Marinescu, Brian Chow, Alexander D Wissner-Gross e Peter Carr. Available in: http://parts.igem.org/Part:BBa_B0032 |
| *strong RBS* | Ribosome biding site with strong affinity for *E. coli* ribosomal 16S RNA. | Registry of Standard Biological Parts (iGEM Foundation): Part BBa_B0034, designed by Vinay S Mahajan, Voichita D. Marinescu, Brian Chow, Alexander D Wissner-Gross e Peter Carr. Available in: http://parts.igem.org/Part:BBa_B0034. |
| *TT (E. coli)* | Natural transcription terminator of *E. coli* RNA polymerase*.* | Registry of Standard Biological Parts (iGEM Foundation): Part BBa_B0010, designed by Randy Rettberg. Available in: http://parts.igem.org/Part:BBa_B0010 |
| *TT (T7)* | Transcriptional terminator of T7 bacteriophage RNA polymerase. | Registry of Standard Biological Parts (iGEM Foundation): Part BBa_K731721, designed by Giacomo Giacomelli e Anna Depetris. Available in: http://parts.igem.org/Part:BBa_K731721. |
| *tetR + lva* | TetR encoding gene. Containing fast degradation tag. | Registry of Standard Biological Parts (iGEM Foundation): Part BBa_C0040, designed by June Rhee, Connie Tao, Ty Thomson, Louis Waldman. Available in: http://parts.igem.org/Part:BBa_C0040. |
| *tevp* | Tobacco Etch Virus protease encoding gene. | (Kapust et al., 2001) |
| *cI434 + lva* | cI434 encoding gene. Containing fast degradation tag. | Registry of Standard Biological Parts (iGEM Foundation): Part BBa_C0052, designed by Maia Mahoney. Available in: http://parts.igem.org/Part:BBa_C0052. |

**Supplementary Table S2** – Detailed description of plasmids assembled in this work.

| Vector name | insert | Insert standardization | Backbone | Origin of replication | Resistance |
| --- | --- | --- | --- | --- | --- |
| pM1A | *pT7_lacO_RBS_eda* | *RFC[23]* | pUC57 | ColE1 | Ampicillin |
| pM2A | *egfp_medium-RBS_cI434_TT(pT7)* | *RFC[23]* | pUC57 | ColE1 | Ampicillin |
| pM3A | *pL-cIO_strong-RBS_TetR_TT(E. coli)_pL-tetO_weak-RBS_tevp_TT(E. coli)* | *RFC[23]* | pUC57 | ColE1 | Ampicillin |
| pM2C | *egfp_medium-RBS_cI434_TT(pT7)* | *RFC[23]* | pSB1C3 | pMB1 | Chloramphenicol |
| pM12C | *pT7_lacO_RBS_eda-egfp_medium-RBS_cI434_TT(pT7)* | *RFC[23]* | pSB1C4 | pMB1 | Chloramphenicol |
| pSOLC | *pT7_lacO_RBS_eda-egfp_medium-RBS_cI434_TT(pT7)_pL-cIO_strong-RBS_TetR_TT(E. coli)_pL-tetO_weak-RBS_tevp_TT(E. coli)* | *RFC[23]* | pSB1C5 | pMB1 | Chloramphenicol |
| pSOLA | *pT7_lacO_RBS_eda-egfp_medium-RBS_cI434_TT(pT7)_pL-cIO_strong-RBS_TetR_TT(E. coli)_pL-tetO_weak-RBS_tevp_TT(E. coli)* | *RFC[23]* | pUC57 | ColE1 | Ampicillin |
| pM3K | *pL-cIO_strong-RBS_TetR_TT(E. coli)_pL-tetO_weak-RBS_tevp_TT(E. coli)* | *RFC[23]* | pSB3K3 | p15A | Kanamycin |

**Supplementary Table S3** – Fluorescence values found for the four conditions tested at DO_600nm =_ 1.5

| **Plasmid** | ***E. coli* strain** | **Max. Fluorescence (A.U.)** | **Mean ± SD** | **p value** ^a^ |
| --- | --- | --- | --- | --- |
| **pSOLA** | **BL21** | 238.7 | 60.01 ± 102.3 | 0.2020 ^b^ |
|  | **Rosetta** | 119.5 | 76.09 ± 32.89 |  |
| **pSOLC** | **BL21** | 180.5 | 87.1 ± 71.52 | 0.4127 ^c^  0.9273 ^d^ |
|  | **Rosetta** | NA | NA | NA |
|  |  |  |  |  |
| **pM12C + pM3K** | **BL21** | 193.6 | 53.28 ± 93.53 | 0.5556 ^e^  0.2303 ^f^  0.3429 ^g^ |
|  | **Rosetta** | NA | NA | NA |

NA = Not available, due to the Cm^R^ phenotype of the Rosetta strain.

^a^ Mann-Whitney test.

^b^ pSOLA (BL21) vs. pSOLA (Rosetta).

^c^ pSOLC (BL21) vs. pSOLA (BL21).

^d^ pSOLC (BL21) vs. pSOLA (Rosetta).

^e^ pM12C + pM3K (BL21) vs. pSOLA (BL21)

^f^ pM12C + pM3K (BL21) vs. pSOLA (Rosetta)

^g^ pSolC (BL21) vs. pM12C + pM3K (BL21)

**Supplementary Table S4** – Primers used in the RT-qPCR experiment

| **Target Gene** | **Sequence (5' - 3')** | **Tm (oC)** | **GC %** | **Amplicon size** |
| --- | --- | --- | --- | --- |
| *eda* | Foward: GGTGCACAGTTCGCAATTAG | 62 | 50 | 123 |
|  | Reverse: GTAGTCCATACCCAGCATCAG | 62 | 52.4 |  |
| *egfp* | Foward: CTTTGCGAGATACCCAGATCATA | 62 | 43.5 | 114 |
|  | Reverse: GTCTTGTAGTTCCCGTCATCTT | 62 | 45.5 |  |
| *cI434* | Foward: GTCATAGATGGCGGTCAGAAG | 62 | 52.4 | 100 |
|  | Reverse:CTTCCACGACAACACCGATAA | 62 | 47.6 |  |
| *tetr* | Foward: GCAGAGCCAGCCTTCTTATTC | 63 | 52.4 | 80 |
|  | Reverse: GCGGACCCACTTTCACATTTA | 63 | 47.6 |  |
| *tevp* | Foward: TCATCATCCGTATGCCGAAAG | 62 | 47.6 | 98 |
|  | Reverse: GTTAGTGGTAACCAGGCAGATAC | 62 | 47.8 |  |
| *16s rRNA* | Foward: TCAAGTCATCATGGCCCTTAC | 62 | 47.4 | 111 |
|  | Reverse: CGGACTACGACGCACTTTAT | 62 | 47.6 |  |


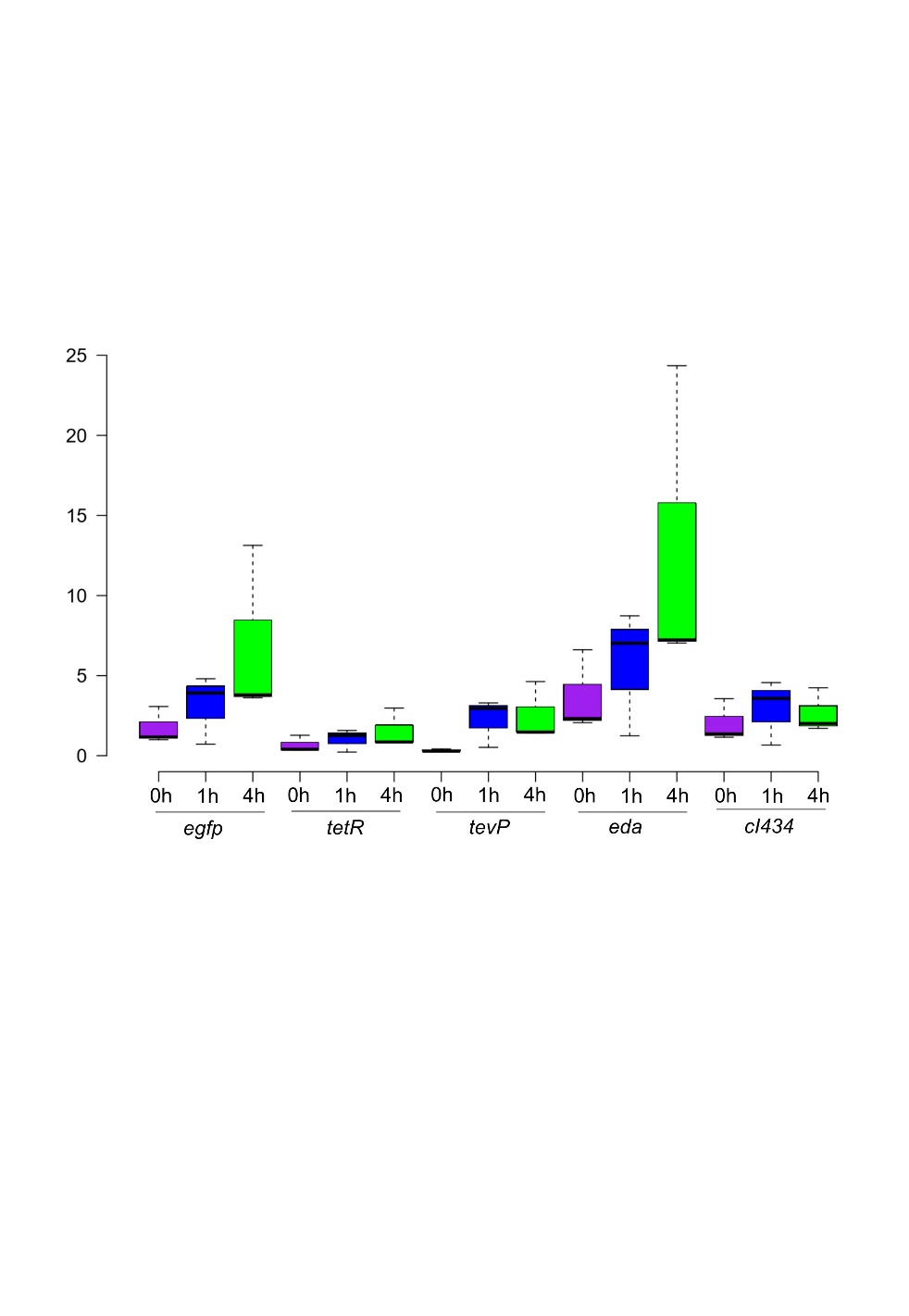

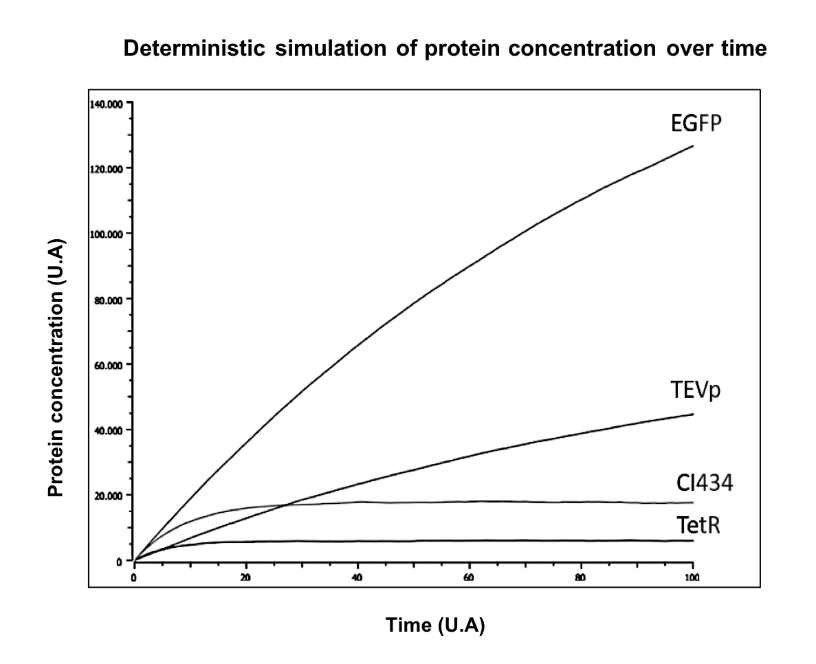


**A**

**Supplementary FIGURE S1. Functioning of the genetic system for controlled intracellular processing of fusion proteins in *E. coli*. A)** Deterministic simulation of protein expression in the genetic circuit using Thinker cell 2.0. Considering that fusion protein (EDA-EGFP) coding sequence is under control of stronger transcription and translation elements than TEVp, it is expected that the latter would accumulate less after IPTG induction. Because cI434 and TetR have LVA tails, which causes fast degradation, it was probable that repressor proteins concentration would remain nearly constant. **B)** Relative expression of the elements composing the genetic system, following IPTG induction (time 0h). Total RNA was extracted at times 0h, 1h, and 4h, and gene expression was evaluated using RT-qPCR, as described in Supplementary Methods, using primers presented in Table S4. 16S rRNA gene was used as reference gene for normalization of relative quantification data. Fold-change was calculated in comparison to the expression level of *egfp* at time 0h. *eda* expression follows a similar pattern of expression when compared to *egfp*, though reaching apparently higher levels (not significant difference: P value = > 0.99 at time 4h; Dunn's post hoc test). This might be due to its upstream position in the transcriptional unit and also due to differences in primer efficiencies between genes. Expression of *tevP* significantly increases at 4h post-induction, when compared to time 0h, but at levels below those achieved for *egfp* and *eda*, as expected. *tetR* shows an unexpected, but non-significant, increase in expression at time 4h, which might be related to an imbalance in expression of the repressor protein cI434.

**B**


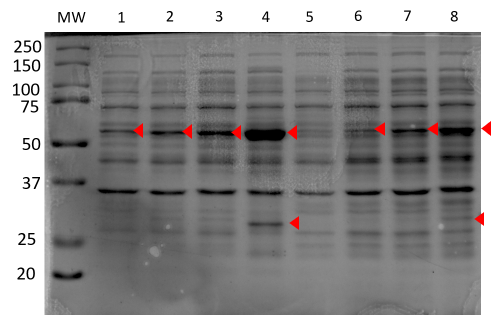


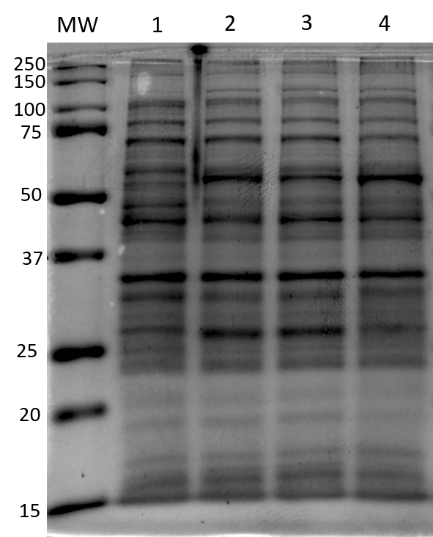


EDA-EGFP

**A**

**B**

EDA-EGFP

EGFP

**C**


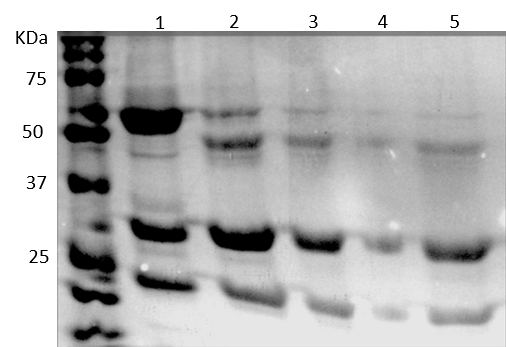

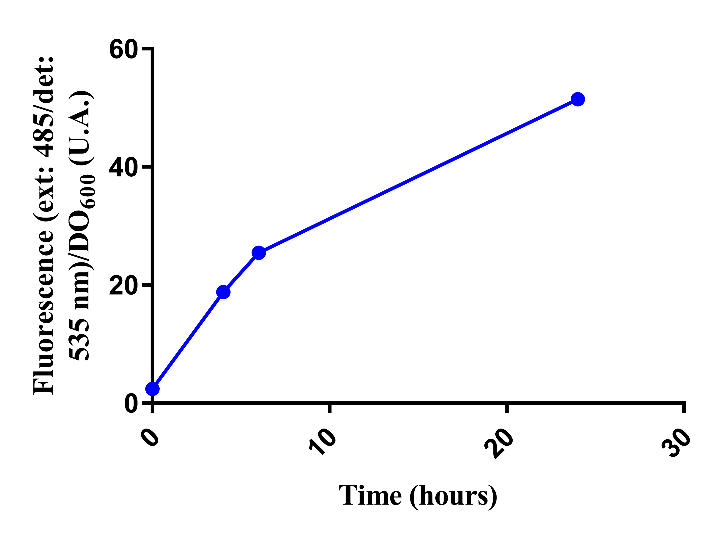


**E**

**D**

**Supplementary FIGURE S2. A)** 12% SDS-PAGE of lysate containing proteins expressed in BL21 (1-4) and Codon-plus (5-8) transformed with pSOLA induced with 0.5 mM of IPTG at OD_600_ 1.5 at 25^o^C. 1 and 5 – 0h; 2 and 6 - 4h; 3 and 7 – 6h; 4 and 8 – 24h. **B)** 12% SDS-PAGE of proteins expressed in Rosetta transformed with pSOLA induced with 0.5 mM of IPTG at OD_600_ 1.5 at 25^o^C. 1-4 lysates obtained respectively 0, 4, 6 and 24 hours of induction. **C)** Fluorescence measurements at 0h, 4h, 6h and 24h of induction at 25 and 37^o^C. **D)** TEV protease was incubated with his-tagged purified protein from pM1.2C and pM3K transformed BL21 culture. *In vitro* cleavage of EDA-EGFP fusion using TEV protease (pro TEV plus-Promega, WI, USA). 1-5 - Aliquots were taken at 0, 1, 2, 3 and 4 hours after incubation with the protease. It could be seen that EDA-EGFP fusion substrate is cleavable by TEV protease and few hours is necessary for almost complete tag removal *in vitro*. 10 U of ProTEV Plus were used per 100 µg of EDA-EGFP. **E)** Measurement of green fluorescence (excitation 485 nm and detection 535 nm) per DO_600_ for 24 hours. Measures were taken from BL21 at 0, 4, 6 and 24 hours of induction.

**Supplementary MATERIALS AND METHODS**

**Cloning Experiments**

JM109 and XL1Blue *E. coli* strains were used in all cloning experiments. These strains were grown overnight and cultivated at 37°C in Luria–Bertani (LB) medium with the right amount of proper antibiotic, when required (100 μg mL^-1^ ampicillin, 34 μg mL^-1^ chloramphenicol, 50 μg mL^-1^ kanamycin). Plasmid recovery was performed using NucleoSpin plasmid mini-prep Kit (Macherey Nagel Co., Düren, Germany). Restriction enzymes and T4 DNA ligase were purchased from Promega (Madison, WI, USA) and used as per the manufacturer’s instructions. DNA segments were isolated by 1% agarose gel electrophoresis in TAE buffer, and purified using the agarose gel DNA extraction kit Illustra GFX PCR DNA and Gel Band Purification (GE Healthcare, Waukesha, WI, USA). Chemically competent *E. coli* cells were transformed by using heat shock method, as follows: bacteria were mixed with the desired plasmid, then cells were cooled down for 30 min., heated at 42^o^C for 1 min. and thawed on ice for further 5 min. Finally, cells were gown in LB media at 37^o^C for 1 h and then plated on LB agar plates containing the right concentration of antibiotic.

**Recombinant EGFP Expression**

BL21 *E. coli* strain was transformed with pSOLC, pSOLA or double transformed with pM1.2C and pM3K. pSOLA was also transformed in Rosetta™ (DE3) (Novagen, Madison, WI) and Codon-plus-RIL strains. After growth for 16 h in LB-Agar plates with the proper antibiotic, one single antibiotic resistant colony of each transformation was gown in shaker flask containing liquid LB media plus antibiotics. They were cultivated to exponential or stationary growth phases (OD_600nm_ range = 0.4-3.0) at 37^o^C, and then 0.5 mM of IPTG was added to induce production of recombinant proteins and temperature was turned to 25^o^C. Aliquots of 1 mL of each culture were recovered at 4 and 24 hours post induction, centrifuged at 11,000 x g and resuspended in 200µL of 10 mM sodium phosphate buffer (pH 8.6).

**Solubility test**

Bacterial pellets were thawed at room temperature, then resuspended in 500 µl of FastBreak™ Cell Lysis Reagent (Promega, Madison, WI, USA). Samples were vortexed for few seconds and incubated in an agitator for 20 minutes. Each sample was sonicated 3 times for 30 seconds at 60 of amplitude. All lysates were centrifuged at 25000xg for 15 minutes and soluble supernatant was collected. Remaining pellets were washed by resuspending it on PBS and then centrifuging at 25000xg for 15 minutes, in order to wash any remaining soluble protein. Finally, insoluble pellets were resuspended in 8M Urea containing buffer.

**Purification of** **His-Tagged EGFP**

His-tagged proteins were purified from 2 mL of bacterial pellets by using MagneHis™ Protein Purification System (Promega, Madison, WI, USA) using the soluble protein protocol. Purified protein was then filtrated through Amicon® (Lexington, MA, USA) Ultra 2 mL Centrifugal Filter (50 kDA cutoff), in order to separate fusion EDA-EGFP from detached EGFP. It was possible to obtain pure EGFP in the eluate after 30 minutes of centrifugation at 7,500 x g. Protein concentration was measured by Qubit™ Protein Assay Kit (ThermoFisher).

**SDS-PAGE and Western-Blot Analysis**

Samples were heated at 100^o^C and diluted with DTT/BME and then analyzed in 15% polyacrylamide gels stained with Coomassie blue. ImageJ software was used to compare the proportions of fused EDA-EGFP and EGFP on purified protein eluate. Proteins run in SDS-PAGE were transferred to nitrocellulose paper in cold buffer for 1 hour at 350 mA. After membrane blocking (PBS, 5% skimmed milk, 3% of tween-20) at 4^o^C overnight, EGFP proteins were detected using eGFP Tag Monoclonal Antibody (Invitrogen, F56-6A1.2.3, 1:4000), Anti-mouse rabbit IgG alkaline phosphatase conjugated antibody (Sigma, 1:400) and BCIP/NBT Color Development Substrate (Promega), using standard Western blot protocols. Western blot images were obtained using ImageQuant LAS 500 series (GE Healthcare).

**Fluorescence and OD_600_ Measurements**

Multiskan™ FC Microplate Photometer was used to measure bacterial density, adjusting wavelength to 600 nm. Fluoroskan Ascent™ Microplate Fluorimeter measured EGFP fluorescence in bacterial cultures, by setting excitation wavelength to 485 nm and emission wavelength to 535 nm.

**RNA extraction and reverse transcription real-time qPCR (RT-qPCR)**

Aliquots of 500 µl of Rosetta culture were collected at 0, 1 and 4 hours of induction with IPTG and mixed with 1 ml of RNAprotect^TM^ Bacteria Reagent (QIAGEN) by vortexing and then incubated for 5 minutes. After that, tubes were centrifuged for 10 min at 10,000 x g. Supernatants were removed by inversion and pellets were stored at -70 ^o^C. RNA isolation was proceeded by using NucleoSpin RNA isolation kit (Macherey-Nagel), as per the manufacturer’s protocol, executing the DNAse treatment step with 95 µl of rDNA for 15 minutes. After RNA isolation, samples were stored at -70 ^o^C.

Total RNA was incubated at 70 ^o^C and then chilled at 4 ^o^C using a thermocycler. Then 200 ng of RNA was mixed with reverse transcriptase mix (GoScript™ Reverse Transcription System - Promega). Total RNA was reverse transcribed in cDNA by using the following cycling conditions: 25 °C, for 5 min; 42 °C, for 1 h; reverse transcriptase inactivation at 70°C for 15 min. qPCR was proceeded by mixing 4µl of each cDNA sample with GoTaq® qPCR Master Mix (Promega, Madison, WI, USA) and 0.2 µM of each primer (sequences available in Supplementary Table 4). Reactions were performed in triplicate. Cycling program was set as follows in CFX96 Touch™ Real-Time PCR Detection System (BioRad): 1 cycle at 95 °C for 2min; 44 cycles of denaturation at 95 °C for 15s, and annealing/ extension at 60°C for 60s; end-point dissociation curve at 60–95 °C. Syber-green fluorescence was analyzed using CFX Manager software (BioRad). Data normalization was done with levels of *16S rRNA* expression.

**REFERENCES**

Correa, A., Ortega, C., Obal, G., Alzari, P., Vincentelli, R., and Oppezzo, P. (2014). Generation of a vector suite for protein solubility screening. *Front. Microbiol.* 5, 1–9. doi:10.3389/fmicb.2014.00067.

Kang, Y. S., Song, J. A., Han, K. Y., and Lee, J. (2015). Escherichia coli EDA is a novel fusion expression partner to improve solubility of aggregation-prone heterologous proteins. *J. Biotechnol.* 194, 39–47. doi:10.1016/j.jbiotec.2014.11.025.

Kapust, R. B., Tözsér, J., Fox, J. D., Anderson, D. E., Cherry, S., Copeland, T. D., et al. (2001). Tobacco etch virus protease: mechanism of autolysis and rational design of stable mutants with wild-type catalytic proficiency. *Protein Eng.* 14, 993–1000. doi:10.1093/protein/14.12.993.

Lentini, R., Forlin, M., Martini, L., Del Bianco, C., Spencer, A. C., Torino, D., et al. (2013). Fluorescent proteins and in vitro genetic organization for cell-free synthetic biology. *ACS Synth. Biol.* 2, 482–489.
